# Household waste sorting practice, and factors associated with sorting practice in Bedelle town, Southwest Ethiopia

**DOI:** 10.1371/journal.pgph.0001288

**Published:** 2023-01-17

**Authors:** Dereje Oljira Donacho, Getachew Befekadu Geneti, Mohammed Reshad Kadir, Gutama Haile Degefa, Mukemil Abdella Fugaga

**Affiliations:** 1 Department of Health Informatics, Mattu University, Metu, Ethiopia; 2 Department of Environmental Health Science and Technology, Jimma University, Jimma, Ethiopia; 3 Department of Pharmacy, Mattu University, Metu, Ethiopia; 4 Department of Public Health, Mattu University, Metu, Ethiopia; 5 Buno Beddele Zone Health Department, Oromia Region, Bedelle, Ethiopia; University of Groningen, NETHERLANDS

## Abstract

Household solid waste generation rate in low-income countries is increasing due to population growth and changes in people’s lifestyles. Sorting waste into categories is an important step in household waste management. However, there is limited information about sorting practices in a low-income setting like Ethiopia. Therefore, this study aimed to assess household solid waste (HSW) sorting practices, and factors associated with sorting practices.A community-based cross-sectional study was conducted from April to May 2021 in Bedelle town. Households were randomly selected, and household heads were interviewed using a pre-tested questionnaire. A total of 209 households were included. The proportion of households that practice waste sorting was calculated. Logistic regression was used to assess the association between HSW sorting practices and associated factors. A P-value of less than 0.05 was declared as significant. The Hosmer and Lemeshow tests were used to check for model fitness.The result of the study shows that the proportion of householders who practiced waste sorting practice in the setting was 21.53%. The sex of the household head, information on sorting benefits, and the availability of private waste collectors for resource recovery were factors in practicing waste sorting at the household level. Accordingly, male-headed households are 88% less likely to practice sorting practice than female-headed households, and those having access to information on waste sorting benefits are 3.68 times more likely to practice sorting, and similarly, households, where private waste collectors are active, are about 4 times more likely to practice waste sorting at the household level than their counterpart. This finding calls on the municipality to create awareness about solid waste sorting practices at the household level, involving both male and female householders in waste management and mobilizing waste collectors at the household level to facilitate effective waste sorting and reuse as sustainable solid waste management options in the town.

## Introduction

Waste is defined as any unwanted or discarded item, whether or not intended for sale or for reprocessing. Household waste includes food waste, paper, cardboard, plastics, textiles, leather, yard waste, wood, glass, metal, and household hazardous waste [1].The amount and composition of household waste could be influenced by different factors such as demography, socio-economic factors, housing type, land use, seasonality, weather and climatic conditions, and the type of waste [2]. Sorting waste at the household level is an essential component of solid waste management. Waste source separation is the collection of different waste fractions at the point of generation, where the waste is sorted and the fractions are collected in separate containers [3]. It is also known as waste separation, waste classification, or waste segregation. Effective sorting practices are a critical point of action for achieving more sustainable waste management practices [4].

Globally, 2.3 billion tons of solid waste are generated in cities annually and this is expected to reach 3.4 billion tons in 2050 [5]. Approximately 62 million tons of solid waste are produced in Sub-Saharan Africa each year; however, waste collection rates in developing countries are less than 70% [6]. The rate of household solid waste in low-income countries is increasing with an increment in population growth and the changes in the lifestyles of people. In Ethiopia, according to a study finding in Addis Ababa city, the estimated amount of waste generated per day is 0.221kg/c and accounts for 71% of the waste generated by households [7]. Similarly, the daily average solid waste generated in Jimma town is 88,000 kg, of which 87% is generated by households, and the per capita generation rate is 0.550.17 kg/capita/day [8].

The existing evidence indicates that the main barriers to household solid waste sorting practice are a lack of containers for separate HSW collection, a lack of public awareness, and a lack of building space for sorting and recycling [9]. The mixed nature of waste generated at the household level makes waste handling and resource recovery difficult. As a result of being dumped on the side of the road or in ditches, a significant amount of household waste ends up in open dumps or drainage systems, endangering both surface and groundwater quality and creating a favorable breeding environment for vectors [10]. Moreover, poor waste management may result in disasters and disease outbreaks. The situation may worsen due to the current urban population growth, rising urbanization, a lack of public education on household waste sorting practices, waste reduction, and recycling programs, and a weak awareness creation campaign.

Household waste sorting practice plays an essential role where waste prevention, recycling, reuse, and recovery are fundamental elements needed for the reduction of solid waste disposed of in landfills [2]. Other works of literature in the country show that the level of waste sorting practice is significantly very low. For instance, the result of a study result from Dessie town shows that about 62.76% of households implement household waste sorting practices regularly [11]. According to the finding of the study conducted in Bahir Dar City, about 23% of households carry out HSW sorting practices [12, 13]. However, there is limited information about the factor associated with sorting. Therefore, this study aimed to assess sorting practice, and associated factors in Bedele town, in South-west Ethiopia. These study results may help urban planners, health workers, and researchers interested in waste management focus on sorting practices as the base for sustainable waste management.

## Methods and materials

### Study area and period

This study was conducted in Bedelle Town, the capital of Buno Bedele zone, Oromia regional state in southwestern Ethiopia, 480 kilometers from Addis Ababa, Ethiopia’s capital city, and located at 8° 27′ North, 36° 21′ East. The population of the town of Bedele is estimated to be 32,168 (16131 male and 16037 female) with 6702 households. The town was designated as the capital of the newly formed Buno Bedele zone in 2017. Administratively, the town is divided into two kebeles. The town has grown rapidly, and urbanization is increasing at an alarming rate, especially since 2017.

### Study design and population

This was a community-based cross-sectional quantitative study design that aimed to assess household solid waste sorting practice and associated factors in Bedele town from April 15 to May 15, 2021. The source population was the households found in the town, and the study population was selected households in each kebele of the town. Household heads or family members who were aged above 18 years and were available at the house during the study period were included, and households that were closed during the revisit were excluded from the study.

### Sample size determination

The number of sample households was determined by using the following formula [14]: $n=\frac{Nz^{2}PQ}{d^{2}(N-1)+z^{2}PQ}$, Where n = sample size of housing units, P = housing unit variable (residential houses), Q = non-residential houses (offices, schools, etc. in terms of % average) = 1-P, N = total number of housing units, Z = standardized normal variable and value that corresponds to 95% confidence interval, d = allowable error (0.05), N = based on information from the municipality of the town, there are about 6,702 legal housing units (N): Out of these, more than 85% (P) are residential and the rest 15% (Q) are for commercial activities, offices, and others. Hence, n = 190, considering a 10% non-response rate, the total sample size was 209 HHs included.

### Sampling technique and procedure

The study area, Bedelle town, has 2 kebeles and 17 sub-villages. A systematized random sampling method was used to ensure homogenous data and sample collection. Each sample household had an equal chance of being included in the study. From the total of 6,702 households in 17 sub-villages of the town, the first kebele has a total of 3,329 households, and we sampled 104; the second kebele has a total of 3,373 households, and we sampled 105, and we have a total of 209 households. To maintain the representativeness of the study, respondents were picked systematically from every "i^th^" household (i = N/n). Where: N = the total number of households in the sampling frame list adopted by the municipality of the town; "n" is the number of sample households that was determined. Finally, after the sample size was identified, a simple random sampling method was employed to draw samples from the target HHs. The first HH was chosen using the lottery method.

A random sampling technique was employed to collect the socio-demographic data from the selected households by using a structured questionnaire. To gate household solid waste collection, sample collectors provide two (2) plastic bags to each of the selected households and the householders were requested to put all types of solid waste in the provided plastic bag. To reduce errors arising from the weight of sampled household waste, the weight of each empty bag was measured before being distributed to the sampled households. The waste collected on the first day was discarded, as it may have been the accumulation of many days’ waste. The sampled household’s proper waste collection status was followed by supervisors and investigators twice per week, and at the end of the seventh day, waste-filled bags were collected and transported to the waste sorting site.

In the waste sorting practice, the amount of waste generated by each household was determined by weighing the collected sample waste. Food wastes, organics, paper and paperboard, plastics, glass, metals, leather and rubber, textiles, wood waste, ash, and other wastes were then separated into 11 categories and placed in pre-weighed plastic bags that were labeled and plastered with predetermined categories. The waste was then sorted into categories and weighed and calculated to determine the net value of the sample component and plastic bag, which was then recorded on sample recording formats.

### Data collection tools and methods

The data collection tools for the study were questionnaires, observation checklists, and household sample waste collection bags. The weight of each empty bag was measured before being distributed to the sampled households. The waste generation at each household was measured on a weight scale and recorded.

### Data processing and analysis

The data was entered using Epi-data version 3.1 [15] and exported to SPSS [16] version 23 for analysis. The descriptive analysis was done. The mean and proportions were calculated and presented using tables. Binary logistic regression was used to assess the association between household solid waste sorting practices and associated factors. Multivariable logistic regression was done for each independent variable, and variables with a p-value of 0.25 were taken as candidates for multivariable logistic regression. Finally, the odds ratio with 95% CI was checked, and a P-value less than 0.05 was used to declare statistical significance. The Hosmer and Lemeshow tests were used to check for model fitness.

### Data quality control

The questionnaire was prepared in English and then translated into the local language (Afan Oromo). A pretest of the questionnaire was carried out on 5% (11 HHs) of the total sample size in the similar town of Kolo korma kebele of Metu town, which is outside of the study area, before the actual data collection period. To reduce errors arising from the sampled household waste, the weight of each empty bag was measured before being distributed to the sampled households. Two days of training were given to data collectors and supervisors on how to handle, measure (weigh), sort, calculate, and record data from collected sample solid waste. Moreover, the completeness and consistency of data were checked daily by the supervisors and the principal investigator with necessary corrections to ensure all the information was properly collected, completed, and coded.

### Operational definitions

"Solid waste" was defined as waste generated at home by residential houses. Furthermore, household solid waste sorting was defined as the separation of household waste into various material streams or categories. The households that practice segregation of household wastes into different categories as per their report, and cross-check the availability of sorted wastes or separate containers for waste streams during observation, which was considered as they practice sorting.

### Ethical clearance

Mattu University’s College of Health Sciences-Department of Public Health provided ethical approval for the study. Before starting the actual study, the Bedele town municipality and local (kebele) leaders were asked for permission to collect data and sample waste, and the willingness of households in the study population was taken into account. Written informed consent was taken before starting the data collection from the head of the household. Throughout the process, personal safety procedures, including personal protective equipment, were used.

## Results

### Socio-demographic characteristics of respondents

A total of 209 households participated in the study, with a 100% response rate. The marital status of the household heads indicated as single, married, divorced, widowed, and separated was 11.48%, 79.90%, 4.31%, 2.87%, and 1.44%, respectively. The mean age of the respondents was 42 years. The majority of the households have a family size of fewer than five members (85%), and 31 (14.8%) have a family size of five or above, with a mean of 4.8. In terms of study participants’ residences, 114 (54.5 percent) live in private houses, while the remaining 96 (45.5 percent) live in rental houses. Furthermore, 173 (82.78 percent) of the respondents live in multi-room houses, while the remainder lives in single-room houses (Table 1).

**Table 1:** Socio-demographic characteristics of respondents in Bedelle town, Southwest Ethiopia, 2021.

Variables		Frequency (%)
Sex household heads	Male	178 (87.6)
	Female	26 (12.4)
Age of Household heads (mean = 42 years)	<20	4(1.91)
	20–29	24 (11.48)
	30–39	87(41.63)
	40–49	61(29.19)
	50–59	19 (9.09)
	60+	14 (6.70)
Family size (mean = 4.83)	less than five	178 (85.2)
	5 and above	31(14.8)
	Primary education (1–8)	30 (14.35)
	Secondary school (9–12)	119 (56.93)
	Above 12^th^ grade	60 (28.71)
Average monthly income of the family	<2500ETB	37(17.7)
	2500–4500	82 (39.23)
	4501–7000	65 (31.10)
	7001–10000	25 (11.95)
Marital Status	Single	24 (11.48)
	Married	167 (79.9)
	Divorced	9 (4.31)
	Other (i.e Widowed:6, separated:3)	9 (4.31)
Availability of land in the yard	Available	122 (58.37)
	Not Available	87(41.63)
Type of the house (Number of rooms)	Single room	36 (17.22)
	Multi-room	173 (82.78)
Housing type (Ownership)	Private	114 (54.5)
	Rental	95 (45.5)

### Household waste generation and sorting practice

The waste generation rate of household solid waste generation rate was about 0.20kg/c/d and 0.87kg/HH/day. Among the generated household solid waste components, about 53.30% are food wastes, 24.08% organics, 2.30% paper, and paper boards, 1.91% plastics, 0.25% glasses, 0.40% metals, 0.51% leather and rubber wastes, 0.44% textiles, 0.19 wood wastes, 15.24% ash and fines, and 1.38% other wastes. Based on the result of this study, the current waste composition in Bedelle town was food waste (about 53.30% by weight), the next major component of the category is organic waste (other than food waste), which accounts for 24.47% by weight, the third major component is ash and fines, which account for about 15.49%, and the rest of all waste categories account for 7.50% by weight of the total waste generated from the study area. Concerning household solid waste sorting practice, it indicated that about (21.53%) of the study households carry out household solid waste sorting practice. According to the result of this study, out of the total respondents, 88 (42.1%) of them reported that they have gotten information about household solid waste sorting benefits from urban health extension workers.

The availability of waste collection containers at the household level was 133 (63.6%). About 61 (29.2%) of the respondents reported having door-to-door private waste collectors in their village, and more than half 124(59.3%) reported having recovered some useful materials from household waste from their previous experience (Table 2).

**Table 2:** Household waste Generation and sorting practice among households in Bedele town, 2021.

Variables		Frequency (%)
HH waste sorting practice at the point of generation	Yes	45 (21.5)
	No	164 (78.5)
Access to information on the benefit of sorting	Yes	88 (42.1)
	No	121 (57.9)
Ever recovered useful resources from waste	Yes	124 (59.3)
	No	85 (40.7)
Availability of door-to-door privet waste collectors in the village (quralew’)	Yes	61 (29.2)
	No	148 (70.8)
Availability of waste collection container in the HH compound.	Yes	133 (63.6)
	No	76 (36.4)

### Factors associated with household solid waste sorting practices

In the current study, many variables were explored to test the association of adjustment of variables using logistic regression to predict variables that were associated with waste sorting practice at the point of generation in households during the crude analysis. In the final model, the sex of the household head, information on sorting benefits, and availability of private waste collectors for resource recovery were factors affecting the practice of waste sorting at the household level (Table 3). Accordingly, male-headed households (AOR/ 95% CI:0.12/0.04–0.67), access to information on waste sorting benefits (AOR/95% CI: 3.68 /1.48–9.15), and availability of private waste collectors in their village (AOR/95% CI: 9.65/3.94–23.63).

**Table 3:** Multivariable analysis of point of source waste sorting practice among households in Beddele, southwest, Ethiopia, 2021.

Variables		SW Sorting Practice		Crude Odds Ratio			Adjusted Odds Ratio		
		Yes (45)	No (164)	COR	CI	P-value	AOR	CI	P-value
Household head sex	Male	27	156	0.07	0.03–0.19	0.001	0.12	0.04–0.39	0.001*
	Female	18	8	1			1		
Information on sorting benefit	Yes	32	56	4.74	2.31–9.76	0.001	3.67	1.48–9.15	0.005*
	No	13	108	1			1		
Ever recover resources from waste	Yes	36	88	3.45	1.56–7.67	0.002	1.81	0.16–19.7	0.626
	No	9	76	1					
Availability of Storage	Yes	37	98	3.28	1.43–7.47	0.005	1.23	0.10–14.7	0.871
	No	8	68	1					
Availability of PWC	Yes	31	30	9.89	4.69–20.8	0.001	9.65	3.94–23.6	0.001*
	No	14	148	1					

Key

* Significant at P-value <0.05, SW: Solid waste, PWC: Privet waste collectors

## Discussion

The study was conducted to assess HSW sorting practices and associated factors among households in Bedele town. The household solid waste generated in Bedele town was 0.87 kg/HH/day and 0.20 kg/c/day. Among other wastes, 53.30% are food wastes, 24.08% are organics (including paper and paperboard), 1.91% are plastics, 0.44% are textiles, and 15.24% are ash and fines. The availability of waste collection containers at the household level was 133 (63.6%), and 61 (29.2%) of the respondents reported having door-to-door private waste collectors in their village. More than half (59.3%) reported having recovered some useful materials from household waste. The daily average generation rate in this study was 0.144 kg/c/d and 0.22 kg/c/d (0.28 in the central zone, 0.17 kg/c/d in the outer zone, and 0.20 kg/c/d in the middle zones), which is consistent with studies conducted in Assosa town [17] and Bahr Dar town [12, 13].

The HSW sorting practice in the study area was about 21.53%. This finding is consistent with the findings conducted in Dessie Town [18]. This finding was lower than the study’s finding from Jigjiga City [19]. The difference could be due to the size and cultural differences between Ethiopia’s east and west.

Household waste sorting was influenced by the gender of the household head, information on the benefits of sorting waste at the point of generation, and the availability of private waste collectors for resource recovery. Accordingly, male-headed households were 88 percent less likely (AOR/95% CI: 0.12/0.04–0.67) to practice sorting the wastes at the point of generation in the household, and households’ access to information on waste sorting benefits was 3.68 times more likely (AOR/95% CI: 3.68/1.48–9.15) than the counterpart to practice, and the availability of private waste collectors in their village made them 9.65 times more likely to practice waste sorting (AOR/95% CI: 9.65/ 3.94–23.63). This finding is consistent with another study in which sorting information was an important factor influencing household participation in waste separation practices at sources [20]. Similarly, the finding from Nur-Sultan City in Kazakhstan, HSW sorting behaviors, indicated that females appeared to be more active in source separation than males [9]. The scientific contribution of this discovery assists waste management institutions in focusing on household waste sorting as the foundation for facilitating collection, recycling, and reuse. The discovery assists policymakers in developing institutional frameworks that are currently lacking in Ethiopia’s emerging towns. Due to the cross-sectional nature of our study, it does not show seasonal variations. Furthermore, there may be external influences on the community’s waste handling behaviors that call for future research.

## Conclusion

This study’s findings show the sorting practice in the setting was very low, which may lead to waste management complications for the urban sanitation sector. Household waste sorting was influenced by the gender of the household head, information on the benefits of sorting waste at the point of generation, and the availability of private waste collectors for resource recovery. This finding calls on the municipality to create awareness about solid waste sorting practices at the household level, involving both male and female households in waste management, and mobilizing waste collectors at the household level to facilitate effective waste sorting and reuse as sustainable solid waste management options in the town.

## Supporting information

S1 Data Data set used in this manuscript. (ZIP)

## Abbreviations

HH: Household
HSW: Household solid waste
MSWM: Municipal Solid Waste Management
USWM: Urban Solid waste management
